# Performance Optimization Design for a High-Speed Weak FBG Interrogation System Based on DFB Laser

**DOI:** 10.3390/s17071472

**Published:** 2017-06-22

**Authors:** Yiqiang Yao, Zhengying Li, Yiming Wang, Siqi Liu, Yutang Dai, Jianmin Gong, Lixin Wang

**Affiliations:** 1 National Engineering Laboratory for Fiber Optic Sensing Technology, Wuhan University of Technology, Wuhan 430070, China; yqyao@whut.edu.cn (Y.Y.); daiyt6688@whut.edu.cn (Y.D.); lxwang@whut.edu.cn (L.W.); 2 Key Laboratory of Fiber Optic Sensing Technology and Information Processing, Ministry of Education, Wuhan University of Technology, Wuhan 430070, China; wangyiming@whut.edu.cn (Y.W.); liusiqi@whut.edu.cn (S.L.); 3 Department of Optical Access Network, Huawei Technologies USA, Santa Clara, CA 95050, USA; jianmin.gong@huawei.com

**Keywords:** optical fiber sensing, weak fiber Bragg grating, high speed, distributed feedback laser

## Abstract

A performance optimization design for a high-speed fiber Bragg grating (FBG) interrogation system based on a high-speed distributed feedback (DFB) swept laser is proposed. A time-division-multiplexing sensor network with identical weak FBGs is constituted to realize high-capacity sensing. In order to further improve the multiplexing capacity, a waveform repairing algorithm is designed to extend the dynamic demodulation range of FBG sensors. It is based on the fact that the spectrum of an FBG keeps stable over a long period of time. Compared with the pre-collected spectra, the distorted spectra waveform are identified and repaired. Experimental results show that all the identical weak FBGs are distinguished and demodulated at the speed of 100 kHz with a linearity of above 0.99, and the range of dynamic demodulation is extended by 40%.

## 1. Introduction

With the practical advantages of small size, light weight, multiplexing capability, and resistance to electromagnetic interference (EMI) [1], fiber Bragg grating (FBG) has received considerable interest in the application of mechanical monitoring [2,3,4]. However, high-frequency vibration, a key parameter of mechanical monitoring, can hardly be measured with FBG, as the FBG demodulation speed which is commonly below a few kHz in conventional demodulation technologies such as grating matching [5,6], charge-coupled device (CCD) array [7], and tunable Fabry-Pérot (F-P) filter [8].

Focused on dynamic detection requirements for high frequency signal, some new demodulation methods have been proposed; a fast FBG sensor system using the Fourier domain mode-locked (FDML) swept laser [9,10] was developed to realize a 60 kHz demodulation speed. However, FDML swept laser is complex, expensive, and instable, which limits its adoption for practical applications. Our research team proposed a 100 kHz differential demodulation method based on dispersion compensation fiber and single mode fiber [11]. However, in order to achieve this performance, an ultra-high-speed oscillator with 80 GHz sampling rate is needed, whose cost exceeds the budget of most sensing applications.

In previous work, we demonstrated a high-speed FBG integrated demodulation system based on a distributed feedback (DFB) laser [12]. With a high speed current-modulated DFB laser and a Field Programmable Gate Array (FPGA) real-time data processing circuit, a demodulation speed of 100 kHz was achieved. It is considered as a promising demodulation method in engineering applications for its simple structure, low cost, high reliability, and real-time response. However, the spectral scanning range of the current modulation is only 1 nm, which seriously restricts its capacity and dynamic demodulation range.

In this paper, we combined the high-speed sensing system with identical weak FBG array, whose wavelength change is typically less than 1 nm. We also designed a waveform repair algorithm to estimate the Bragg wavelength of an FBG whose reflection spectrum is only partially detected, and hence broadens the dynamic demodulation range of the FBG sensors. The experimental setup succeeded in demodulating multiple weak FBG sensors at 100 kHz.

## 2. Experimental Setup and Principle

**Figure 1:**
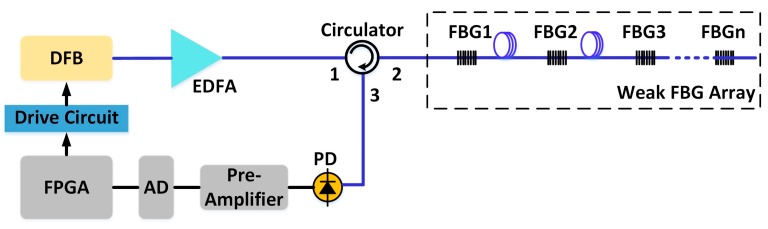
Schematic of a high-speed weak fiber Bragg grating (FBG) interrogation system based on a distributed feedback (DFB) laser.

The schematic diagram of the interrogation system is shown in Figure 1. The DFB laser with a 20 mA threshold is driven by a 100 kHz 20–150 mA sawtooth wave current. The current tuning coefficient of the DFB laser is about 8 pm/mA, then the wavelength scanning range is over 1 nm.

**Figure 2:**
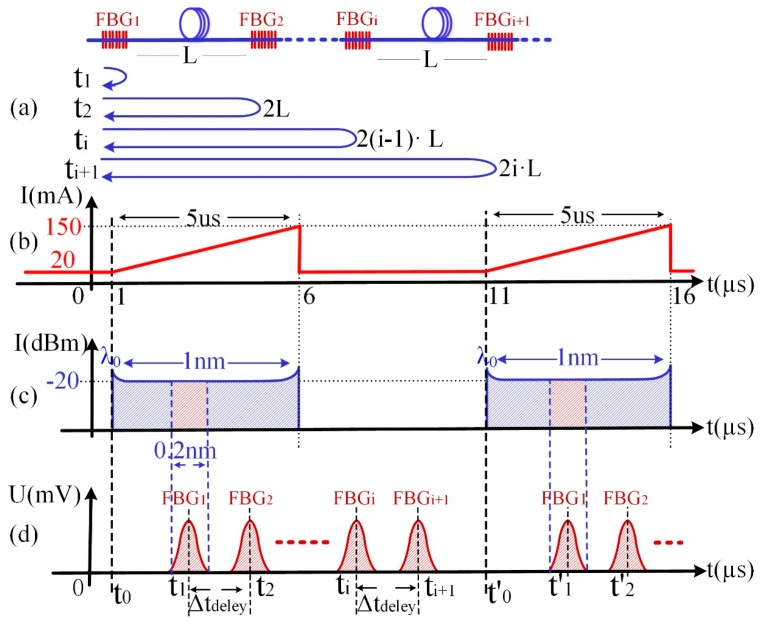
Principle of FBG distinction and demodulation. (**a**) The arrangement of FBGs and the received times of each reflection peaks; (**b**) Driving signal of the DFB laser; (**c**) The output of the DFB laser; (**d**) The reflected waveform of FBGs received by the photodetector (PD).

**Figure 3:**
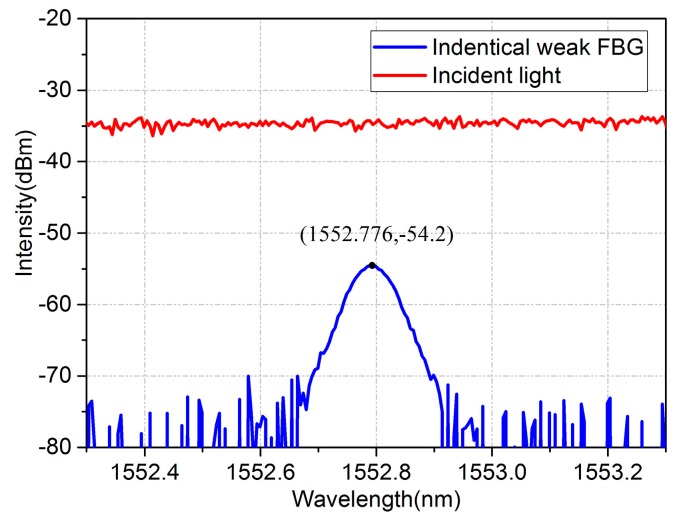
The spectrum of the weak FBG.

After being amplified by an erbium-doped fiber amplifier (EDFA), the swept laser pulses illuminate the identical weak FBG array through an optical circulator. Each grating reflects part of the light (i.e., red region in Figure 2c), and all the reflected light passes the same circulator and is received by a photodetector (PD). Signals from different gratings are separated in the time domain on account of the high scanning speed of the laser and the transmission delay among the gratings, as sketched in Figure 2d. Pre-amplifier, analog-to-digital converter (ADC), and FPGA are used to amplify, sample, and process the serial data from the PD, respectively. A typical spectrum of the weak FBG is shown as Figure 3. The peak reflected intensity from the weak FBG is about −54.2 dBm under −34 dBm incident light. Therefore, the reflectivity of the weak FBG is only 1%, and the reflection spectra of all FBGs have similar 3-dB bandwidths of about 200 pm. Meanwhile, the wavelength difference in the weak FBG array has been controlled within 50 pm in the practical system. Therefore, it will avoid the disunity of the demodulation on the sensing array because when the spectra of identical FBGs move to the margin of the demodulation range, some FBGs with excessive wavelength difference might have already moved out, causing the disunity of the demodulation on the sensing array.

The distance between two neighboring gratings, namely FBG*_i_* (*i* = 1, 2,…, *n*) and FBG*_i_*_+1_, is fixed as the spatial resolution *L*. As shown in Figure 2a, the time delay Δ*t_delay_* between *t_i_* and *t_i_*_+1_ can be calculated as:

(1) $$\Delta t_{delay}=t_{i+1}-t_{i}=\frac{2L\times n_{eff}}{c}$$

where *c* represents the light speed in the vacuum, and *n_eff_* is the effective refractive index of the single mode optical fiber. Meanwhile, the wavelength-to-time mapping is realized in the optical domain by using a DFB laser that has a fast wavelength scanning speed. Therefore, the reflected spectrum of every FBG is transferred to a temporal waveform and separated with an interval of Δ*t_delay_* in the time domain. The center wavelength of the FBG reflected spectrum can be calculated by the following equation:

(2) $$\lambda_{FBGi}=V_{s}\times \left[t_{i}-\left(i-1\right)\Delta t_{delay}\right]+\lambda_{0}$$

where *V_s_* (nm/s) is the wavelength scanning speed of DFB, and *λ*_0_ is the initial wavelength at the beginning of the scanning. Nevertheless, the non-linear problem of the DFB wavelength-swept laser would change the *V_s_* which will influence the accuracy of the wavelength results. Therefore, we have calibrated and calculated the wavelength-swept speed in a segmented manner to reduce the negative influence from the sweep nonlinearity [12].

Besides, in order to fully distinguish all FBGs’ spectra in the time domain, and to avoid the waveform overlap of the respective 200-pm, 3-dB bandwidths, a spatial interval *L* of 100 m is set in the current system to ensure the sufficient Δ*t_delay_*. Since the wavelength inconsistency from FBGs will also affect the spatial interval, it might require a larger space interval to avoid the waveforms overlap for the FBG fabrication uncertainty. Thus, the wavelength differences of FBGs are controlled within 50 pm in the current system.

Therefore, in this distributed identical weak FBG sensing system, the wavelength information of all the FBGs can be distinguished and acquired at a high repetition rate.

## 3. Incomplete Waveform Repair Algorithm

**Figure 4:**
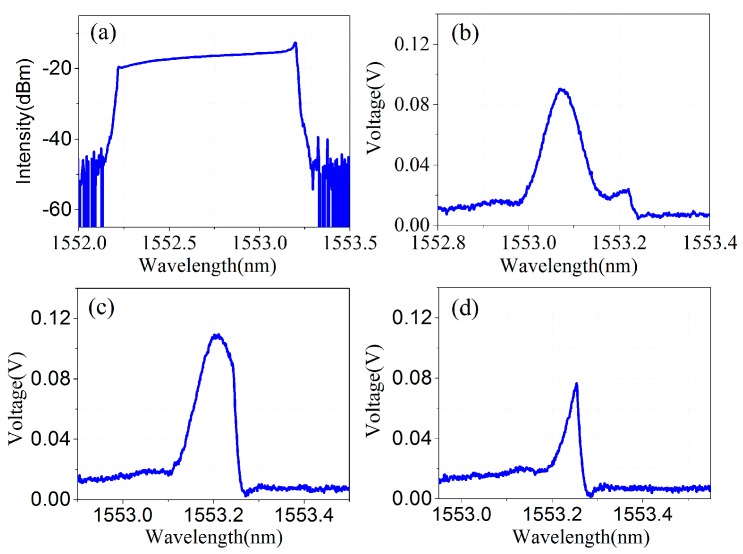
Three types of reflected waveforms of FBG: (**a**) Scanning spectrum of the DFB laser; (**b**) Complete waveform; (**c**) Incomplete waveform type I; (**d**) Incomplete waveform type II.

As shown in Figure 4a, the swept DFB laser has a limited scanning range of 1 nm. In practice, three types of reflected waveforms of FBGs would be obtained: complete waveform, incomplete waveform type I, and incomplete waveform type II, as sketched in Figure 4b–d. In incomplete waveform type I, a small portion of the reflection spectrum of the FBG is out of the laser's scanning range, while the reflection peak remains in the range. In incomplete waveform type II, a large portion of the reflection spectrum including the reflection peak is out of the scanning range. The reflected wavelength of these incomplete waveforms cannot be demodulated by an ordinary Gaussian curve-fitting algorithm [13]. Therefore, in our original demodulation system, considering that the adopted FBG has 200-pm, 3-dB bandwidths and that the incomplete waveform will appear symmetrically in the scanning range of 1 nm, the incomplete waveform type II occupied nearly a 200-pm wavelength range, leaving only an 800-pm dynamic demodulation range left. An efficient incomplete waveform repairing algorithm is proposed in this section.

**Figure 5:**
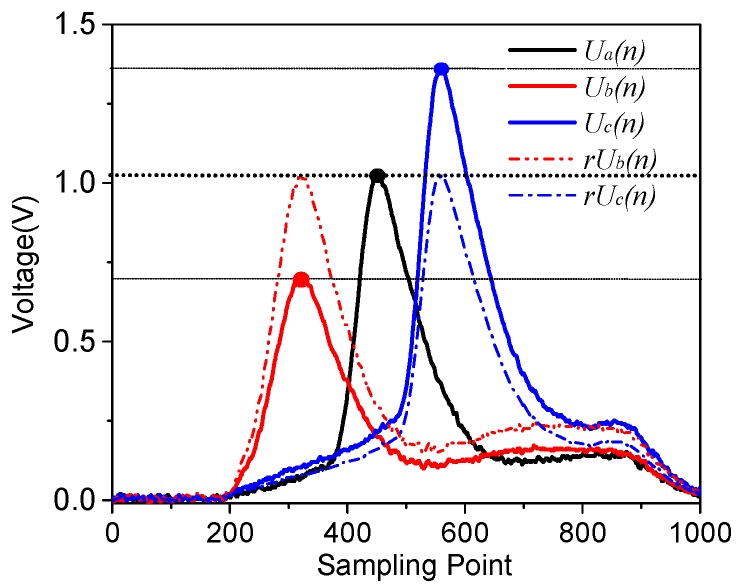
Normalization process of one FBG at different wavelengths.

The incomplete waveform repairing algorithm is based on FBG waveform characteristics, such that its spectral shape *U*(*n*) acquired by the system does not change, but the amplitude of the received waveform will be a little bit different, which is caused by the non-flat-intensity of the wavelength-swept light source and the transmission loss disturbance in the FBG sensor network. As shown in Figure 5, the system has acquired the waveforms of one FBG with wavelength shift, and their peak count is found as a, b, c. It can be seen that their waveforms *U*_a_(*n*), *U*_b_(*n*), *U*_c_(*n*) have different amplitudes.

Their similarities are calculated as follows:

(3) $$\left\{ \begin{array}{l} r\left[U_{a}\left(n\right),rU_{b}\left(n\right)\right]=98.59\%\\ r\left[U_{a}\left(n\right),rU_{c}\left(n\right)\right]=99.77\%\\ r\left[rU_{b}\left(n\right),rU_{c}\left(n\right)\right]=97.85\% \end{array}\right.$$

**Figure 6:**
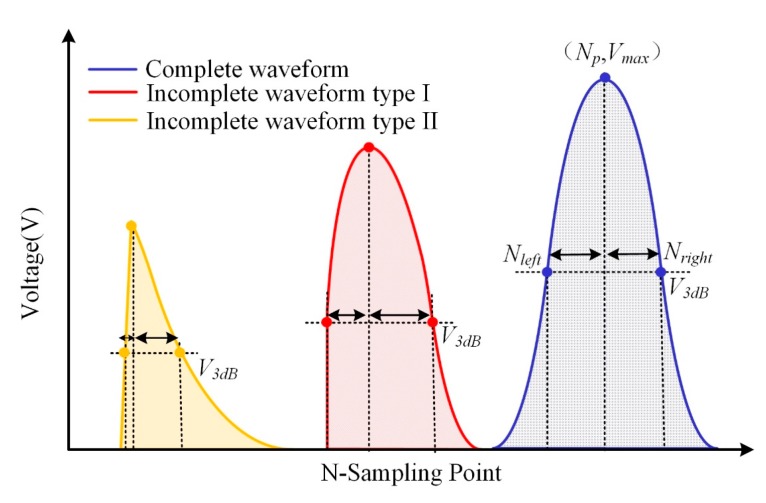
The identification of waveform types.

Their similarities are more than 97%, which means that their original waveform *U*_b_(*n*), *U*_c_(*n*) could be reconstructed under a normalization process by the feature points and the reference waveform *U*_a_(*n*). Therefore, based on this principle, the incomplete waveform can be repaired by a complete waveform as well. The processing procedures are as following:

First, at the initial stage, a waveform type identification based on the waveform symmetry principle is designed to judge the types of waveform. As illustrated in Figure 6, the maximum intensity point of the waveform is marked as (*N_p_*, *V_max_*), and two points of *V*_3*dB*_ are denoted as (*N_left_*, *V*_3*dB*_) and (*N_right_*, *V*_3*dB*_). Then the symmetry distortion ratio *R_sd_* is obtained:

(4) $$R_{sd}=\frac{\max\{|N_{left}-N_{p}|,|N_{right}-N_{p}|\}}{\min\{|N_{left}-N_{p}|,|N_{right}-N_{p}|\}}$$

It can be seen that, with the gradual loss of the wavelength data, the symmetry distortion ratio increases. Therefore, according to the calibrated range of the value of *R_sd_*, we can judge the type of the waveforms to be an complete waveform, incomplete waveform type I, or incomplete waveform type II.

Secondly, we store one complete waveform data for its respective FBG as a reference waveform *U_r_*(*n*) to do the normalization. For incomplete waveform type I, it still has its peak point which can be set as the feature point. Therefore, it is simpler to do the normalization processing from the *U_r_*(*n*) based on the coordinate difference of their peak points.

**Figure 7:**
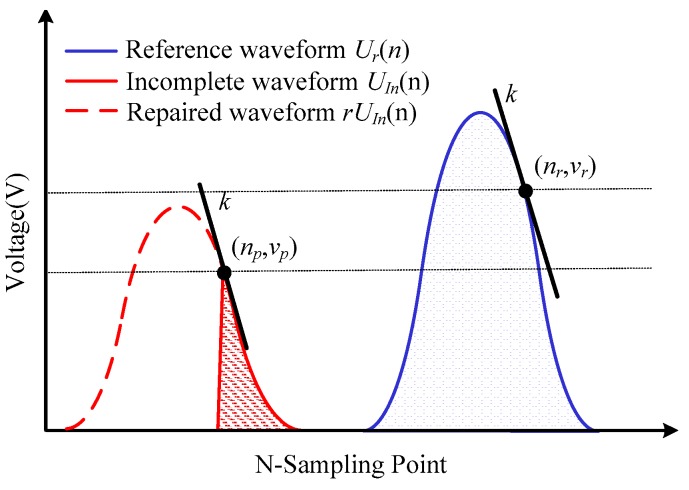
Incomplete waveform repair.

For incomplete waveform type II, as showed in Figure 7, we set the peak point (*n_p_*, *v_p_*) of the acquired incomplete waveform *U_In_*(*n*) as the feature point. Based on the effective waveform data near this peak point, we could calculate the slope k at this position of the incomplete waveform. However, type II has already lost its real peak point and k is no longer zero, which makes it more complex to figure out the corresponding feature point with the same change rate on *U_r_*(*n*).

Therefore, a rapid way is proposed to solve this problem, in which we simplify the reference waveform *U_r_*(*n*) to Gaussian function *U_gr_*(*n*) in an approximate way:

(5) $$U_{gr}\left(n\right)=V_{max}\exp\left[-4In2{\left(\frac{n-N_{p}}{N_{left}-N_{right}}\right)}^{2}\right]$$

where, *V_max_*, *N_p_* and *N_left_* − *N_right_* have already been acquired in the waveform type identification method. Through this Gaussian function *U_gr_*(*n*), we carry out a fast derivative operation of *U′_gr_*(*n*) and *U″_gr_*(*n*) to figure out the only feature point (*n_r_*, *v_r_*) with the same slope k. After acquiring the feature points (*n_p_*, *v_p_*) and (*n_r_*, *v_r_*), as depicted in Figure 7, a normalization process by the stored reference waveform *U_r_*(*n*) can be conducted to acquire the repaired waveform *rU_In_*(*n*):

(6) $$rU_{In}\left(n\right)=\frac{v_{r}U_{r}\left(n\right)}{v_{p}}-\left(n_{r}-n_{p}\right)$$

At last, the system will calculate the similarity of the *rU_In_*(*n*) and *U_In_*(*n*). If the similarity of the overlapping part reaches 97%, a Gaussian peak-seek algorithm [13] will be used to demodulate the center wavelength of the repaired waveform for higher accuracy. When the overlapping part is lower than 97% or the spectrum completely moves out of the detection bandwidth, the waveform cannot be repaired. In order to reduce the occurrence of this situation in practical engineering applications, the working temperature of the DFB laser can be adjusted to match the scanning wavelength center with the wavelength of the FBGs to reserve enough dynamic range.

Moreover, this repairing algorithm is universal for both incomplete waveform type I and type II, and it suggests a very efficient way to realize in our FPGA system, which makes the FBG waveform repairing possible to expand the dynamic demodulation range in practical engineering applications.

## 4. Experiments and Discussion

### 4.1. Temperature Experiment

**Figure 8:**
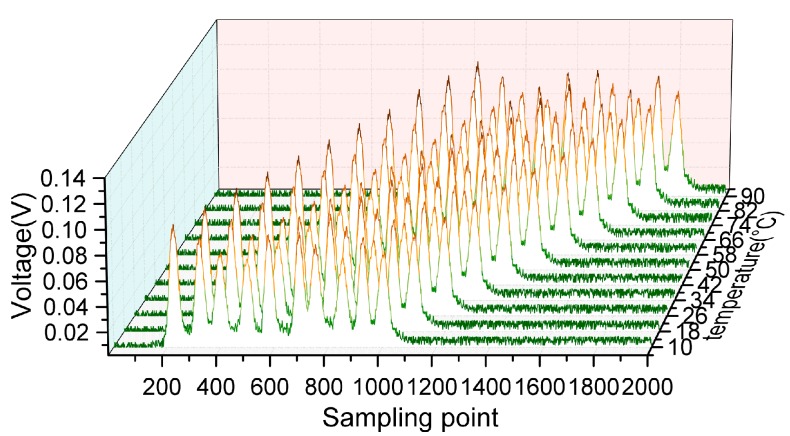
Reflected waveforms from 10 FBGs at different temperature.

We fabricated 10 identical weak FBGs (FBG#1 to FBG#10) with about a 100-m gap along one optical fiber, and placed them into a thermostat. The center wavelength of FBGs are around 1552.7 nm, and the DFB scans from 1552.6 nm to 1553.6 nm by applying a fixed modulation current and adjusting the internal temperature. Then, we changed the thermostat temperature from −10 °C to 110 °C with a step of 2 °C. In Figure 8, reflected waveforms of FBGs within one complete scanning period are acquired by the demodulation system at 200 MHz sampling rate and 100 kHz repetition rate. The sampling point on the *X* axis are the counts of the time, and it can be intuitively seen that 10 identical weak FBGs are separated in the time domain and red shifted with the increase of temperature. Figure 8 demonstrates that the system has a multiplexing capability of more than 10 FBGs. 

**Figure 9:**
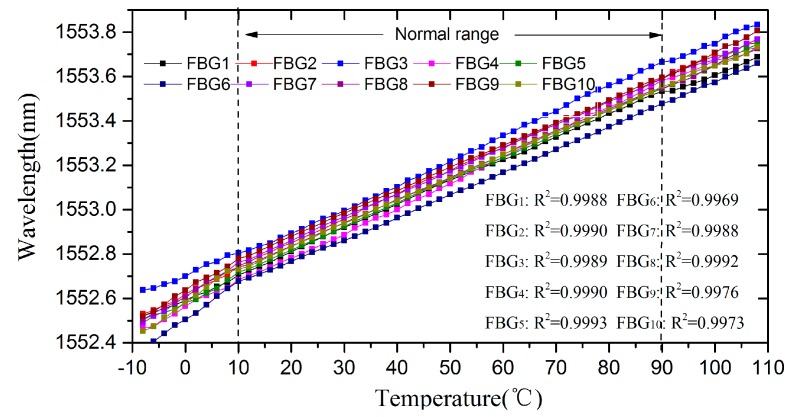
Monitor wavelength shift sensing character of the FBGs in temperature measurement.

When the temperature dropped to 10 °C and rose to 90 °C, the reflection waveforms of FBGs became incomplete. We used the waveform repairing method to demodulate the center wavelength at this temperature range. Therefore, the effective waveforms of FBGs can be demodulated from −8 °C to 108 °C. Figure 9 plots the demodulation results of all FBGs where the R-Squares are 0.9988, 0.9990, 0.9989, 0.9990, 0.9993, 0.9969, 0.9988, 0.9992, 0.9976, and 0.9973 in this whole temperature range. It can be seen that the demodulation linearity decreases in the range of below 10 °C and above 90 °C as the incompleteness of the FBG waveform increases. Nevertheless, with an accepted accuracy for practical application, the demodulation range has evidently expanded to the range of −8–10°C and 90–108 °C.

The temperature experiment demonstrates that the demodulation system capacity is significantly improved for a quasi-distributed FBG demodulation, and the incomplete waveform repairing algorithm can be used to expand the dynamic demodulation range for FBGs. Compared with the ordinary peak-search algorithm in the original system, which only has an 800-pm dynamic demodulation range, the demodulation range is expanded to 1160 pm which is more than a 40% increase for higher capacity FBGs.

### 4.2. Vibration Experiment

We carried out a high-speed vibration experiment to verify the 100 kHz demodulation speed of the system. Specifically, a weak FBG was encapsulated as an accelerometer [14], whose architecture consists of an integrative matrix with base, flexible gemels with inertia masses, and an FBG adhered to the bilateral grooves. By using the sequential quadratic programming method, the structural parameters of this FBG accelerometer were designed to reach the highest eigenfrequency of 3.6 kHz, which meets the requirement of this high-frequency vibration experiment. Then we installed it on a vibration test bench built by a Danish B&K instrument which has a maximum vibration frequency of 4 kHz.

In the experiment, we drove the FBG accelerometer by a 4 kHz sine vibration signal and demodulated the reflection signal in our demodulation system. In order to verify the 100 kHz demodulation speed, we analyzed the sampling points in the time domain and the harmonic component in the frequency domain.

**Figure 10:**
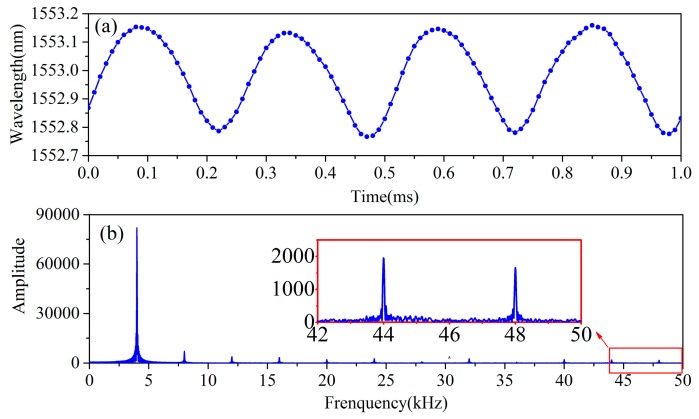
The results of the vibration experiment: (**a**) Distribution of demodulation in the time domain; (**b**) Complete frequency analysis of the demodulation result.

Figure 10a shows the demodulation result in the time domain. In the 4 kHz vibration demodulation, there are 25 sampling points in each vibration cycle, and each sampling interval is 0.01 ms, which demonstrates that the demodulation speed has reached 100 kHz. Furthermore, in Figure 10b, we can obtain the complete frequency distribution of the basic frequency and the harmonic frequencies within 50 kHz of the vibration signal.

## 5. Conclusions

With a focus on the deficiencies of the high-speed demodulation system based on a DFB laser, this paper proposes two methods to improve its performance. First, weak identical FBGs are used to break the limitation of demodulation capacity. Secondly, an FBG reflection waveform repair algorithm is designed to increase the range of dynamic demodulation for FBGs. The experimental results are conducted to demonstrate the effectiveness of this performance optimization design. While reserving the advantages of 100 kHz high-speed demodulation, all of the identical weak FBGs are distinguished and demodulated with 0.99 linearity. Additionally, the dynamic demodulation range is expanded by 40% over the original system. Further improvements in laser performance, such as the nonlinear scanning, wavelength scanning speed and spatial resolution, and the multiplexing capacity of the interrogation system can be expected in the future.
